# Incidence and risk factors for postoperative pneumonia following surgically treated hip fracture in geriatric patients: a retrospective cohort study

**DOI:** 10.1186/s13018-022-03071-y

**Published:** 2022-03-24

**Authors:** Yunxu Tian, Yanbin Zhu, Kexin Zhang, Miao Tian, Shuhui Qin, Xiuting Li, Yingze Zhang

**Affiliations:** 1 Department of Orthopaedic Surgery, The Third Hospital, Hebei Medical University, No. 139 Ziqiang Road, Shijiazhuang, 050051 People’s Republic of China; 2 Hebei Bone Research Institute, Key Laboratory of Biomechanics of Hebei Province, Shijiazhuang, 050051 Hebei People’s Republic of China

**Keywords:** Hip fracture, Epidemiology, Risk factors, Geriatric population, Postoperative pneumonia

## Abstract

**Objective:**

Large sample investigations for risk factors for pneumonia in elderly patients after hip fracture surgeries are lacking. The purpose of this study is to determine the incidence and risk factors for postoperative pneumonia in geriatric patients following hip fracture operations.

**Methods:**

A retrospective study of incidence and risk factors in a tertiary referral center between 2016 and 2020 was conducted. Geriatric patients who developed postoperative pneumonia after surgeries of hip fracture during hospitalization stay were defined as cases and those without as controls. Multivariate logistic regression model was used to evaluate risk factors for postoperative pneumonia.

**Results:**

This study included 3147 patients, and 182 developed postoperative pneumonia, denoting the rate of 5.8%. In the multivariate analyses, age (OR 1.04; 95% CI 1.02–1.06), sex (males) (OR 2.27; 95% CI 1.64–3.13), respiratory disease (OR 3.74; 95% CI 2.32–6.04), heart disease (OR 1.68; 95% CI 1.14–2.47), cerebrovascular disease (OR 1.58; 95% CI 1.11–2.27), liver disease (OR 2.61; 95% CI 1.33–5.15), preoperative stay (OR 1.08; 95% CI 1.05–1.11) and general anesthesia (OR 1.61; 95% CI 1.15–2.27) were identified as independent risk factors for postoperative pneumonia.

**Conclusions:**

This study identified several risk factors for pneumonia in geriatric patients after hip fracture operations, providing a viable preventive strategy for optimizing clinical conditions for reduction of postoperative pneumonia.

## Introduction

Pneumonia is one of the most prevalent complications in older adults following surgeries of hip fracture, with an incidence from 5.1 to 14.9% [1–3]. As the number of hip fracture procedures has been consistently increasing during the past decades in the worldwide [4], the detrimental effect of postoperative pneumonia makes it a significant concern, for either healthcare system or social supporting system. Epidemiologic evidences showed postoperative pneumonia substantially increased the 30-day mortality to be 27–43%, prolonged the hospital stay by 56% and increased the risk of readmission by eightfold [5–7]. The older age, coexistence of multiple comorbidities or markedly reduced organ function reservation, trauma from fracture and operation, prolonged immobilization of limbs after injury, and decreased immune and pulmonary functions contributed to these adverse outcomes [8–10]. The most cost-effective method was to identify the commonly but possibly easily neglected risk factors, especially those modifiable, thereby which early targeted measures can be applied to reduce the occurrence of postoperative pneumonia.

During the past decade, scholars have made numerous attempts on investigation of potential risk factors for postoperative pneumonia following surgically treated hip fracture in geriatric patients, such as sex, age, body mass index, chronic obstructive pulmonary disease, brain injury, smoking history, hypoproteinemia, anemia, number of comorbidities, chronic kidney disease, American Society of Anesthesiologists score ≥ III, functional status, time to surgery and some laboratory biomarkers [5, 10–12]. However, the above studies have some deficiencies that limit the generalization of their results or conclusions, such as limited sample size, inadequate adjustment of confounding factors, or too high selectively population included. Furthermore, most of these studies were from Western countries. Due to the fact that most hip fracture patients in China had to wait for several days, generally 3–7 days, to receive the surgeries, which significantly differed from the recommended early surgical intervention within 24–48 hs [13]. Consequently, the results regarding the epidemiologic characteristics of postoperative complications may also be different. Additionally, the prevalence of osteoporosis directly related to hip fracture, the age at which hip fracture occurred, or prevalence of comorbidities et al differed between Chinese and Western population [14–16]. Therefore, the results available from the western studies might be less generalizable to Chinese population. However, by far few studies in large sample have been focused on this issue.

Given that, we conducted this study, with aims: first, to determine the incidence of postoperative pneumonia following hip fracture surgeries in a Chinese geriatric cohort during the hospitalization stay and second to investigate the risk factors for postoperative pneumonia.

## Materials and methods

This was a retrospective study, conducted in a single-center tertiary referral and university-affiliated hospital (The Third Hospital, Hebei Medical University) between January 2016 and December 2020. The inclusion criteria were patients aged 60 or older presenting with acute hip fracture caused by low-energy injury mechanism and definitely undergoing orthopedic surgery by arthroplasty or osteosynthesis. Exclusion criteria were medium- or high-energy fractures, old fractures (≥ 21 days from initial injury to surgery), pathological fractures, multiple fractures or polytrauma, conservative treatment, revision surgery, re-operations for any reasons, chronic usage of immunosuppressants such as corticosteroids, preoperative existence of pneumonia or respiratory tract inflammation, pre-fracture hip joint functional dependence, death for any cause during hospitalization or patients with incomplete data. The development of postoperative pneumonia was investigated by researchers in the medical records from the day after the surgery to hospital discharge. All data were double entered and cross-checked in order to reduce possible error, and any discrepancies were solved through a consensus via discussion.

### Definition of pneumonia

Diagnosis of pneumonia was based on the American Thoracic Society guidelines for healthcare-associated pneumonia and other online materials [5, 17, 18], based on the following criteria: new and/or progressive and persistent respiratory symptoms such as coughing and purulent secretions, fever or hypothermia (body temperature > 38 °C or body temperature < 36 °C), lung consolidation and/or moist rale confirmed by physical examination, laboratory examination suggesting leukocytosis or leukopenia (white cell count > 10 × 10^9^/L or white cell count < 4 × 10^9^/L), positive blood cultures or sputum sample.

### Variables of interest

Variables of interest included sex, age, living place (rural or urban), body mass index (BMI, categorized as 18.5–23.9, < 18.5, 24.0–27.9 or ≥ 28.0 kg/m^2^), hypertension, diabetes mellitus, respiratory disease heart disease, cerebrovascular disease, liver disease, renal disease, tumors, cigarette smoking, alcohol drinking, fracture type (femoral neck or intertrochanteric), previous surgical history. The surgery-related variables included preoperative stay (from fracture to operation), surgical duration, intraoperative bleeding, intraoperative blood transfusion, procedure (arthroplasty or osteosynthesis), American Society of Anesthesiologists (ASA, categorized as either I–II or III–IV) classification, anesthesia (general or local). Laboratory variables included total protein (TP), serum total cholesterol (TC), triglyceride (TG), white blood cell (WBC), lymphocyte (LYM), red blood cell (RBC), hemoglobin (HGB), hematocrit (HCT), platelet (PLT).

### Statistical analysis

Statistical analysis was carried out using SPSS26.0 (IBM, Armonk, NY, USA). The incidence of postoperative pneumonia was calculated by dividing the number of patients developing pneumonia during hospitalization by the all included patients. The mean ± standard deviation was used to express the continuous variables with normal distribution, and the medians (interquartile range) were used to express the continuous variables with non-normal distribution, following the Shapiro–Wilkes test for normality and followed by t test or Whitney U test, respectively. The number with percentage was used to express the categorical variables, and between-group difference was detected by Chi-square or Fisher's exact test. *P* values < 0.05 were deemed statistically significant. Risk factors tested with significance of *P* < 0.10 in the univariate analyses were included in the multivariate logistic regression analysis to identify the independent risk factors for pneumonia, using backward stepwise mode. Odd ratio (OR) and 95% confidence interval (95% CI) were used to indicate the correlation strength. *P* < 0.05 was considered as significant. The goodness of fit of the final logistics regression model was examined by Hosmer–Lemeshow test, with *P* > 0.05 indicating the acceptable result.

## Results

**Fig. 1:**
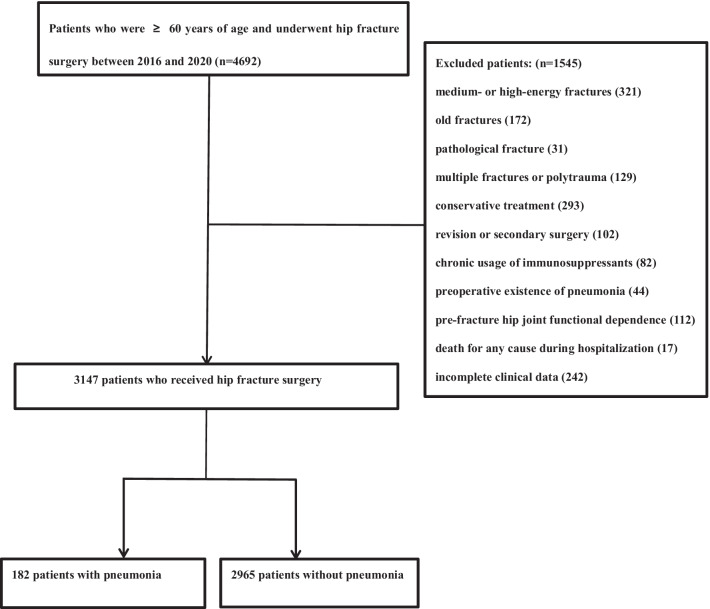
The flowchart showing the selection of research participants

During the study period, a total of 4692 patients (≥ 60 years) were diagnosed with hip fracture, and 1545 cases were excluded due to: medium- or high-energy fractures (321), old fractures (172), pathological fractures (non-osteoporotic fractures) (31), multiple fractures or polytrauma (129), conservative treatment (293), revision or secondary surgery (102), chronic usage of immunosuppressants (82), preoperative existence of pneumonia (44), pre-fracture hip joint functional dependence (112), death for any cause during hospitalization (17), and incomplete clinical data (242) (Fig. 1). A total of 3147 patients were included and 182 were found to have pneumonia, suggesting a cumulative incidence of 5.8%.

**Fig. 2:**
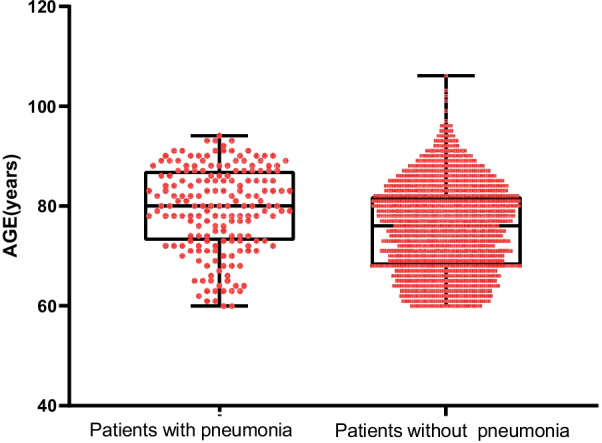
Box plots showing patients with and without pneumonia according to age distribution

Compared with non-pneumonia patients, those with pneumonia had a significantly older age (79.3 ± 8.5 vs. 75.7 ± 8.8) (Fig. 2), more prevalent respiratory disease (15.4% vs. 3.5%, *P* < 0.001), heart disease (26.4% vs. 13.4%, *P* < 0.001), higher proportion of intraoperative blood transfusion (30.2% vs. 23.0%, *P* = 0.026), prolonged preoperative stay (7.5 ± 6.1 vs. 5.3 ± 3.8, *P* < 0.001) and a higher ASA score of III–IV (59.3% vs. 47.0%, *P* = 0.001) (Table 1). Also, the sex (males), cerebrovascular disease, liver disease, renal disease, cigarette smoking, alcohol drinking, fracture type, previous surgical history, anesthesia (general), WBC, PLT were significantly associated with postoperative pneumonia.

**Table 1:** Univariate analysis of variables between pneumonia and non-pneumonia patients

Variables	Pneumonia (n = 182)	Non-pneumonia (*n* = 2965)	*P*
Sex (males)	97 (53.3)	941 (31.7)	< 0.001
Age (≥ 60 years)	79.3 ± 8.5	75.7 ± 8.8	< 0.001
Living place (rural)	87 (47.8)	1577 (53.2)	0.158
BMI (Kg/m^2^)			0.112
18.5–23.9	125 (68.7)	1861 (62.8)	
< 18.5	7 (3.8)	136 (4.6)	
24.0–27.9	33 (18.1)	756 (25.5)	
≥ 28.0	17 (9.3)	212 (7.2)	
Hypertension	105 (57.7)	1574 (53.1)	0.227
Diabetes mellitus	52 (28.6)	708 (23.9)	0.151
Respiratory disease	28 (15.4)	105 (3.5)	< 0.001
Heart disease	48 (26.4)	398 (13.4)	< 0.001
Cerebrovascular disease	56 (30.8)	492 (16.6)	< 0.001
Liver disease	12 (6.6)	79 (2.7)	0.002
Renal disease	17 (9.3)	153 (5.2)	0.015
Tumors	4 (2.2)	79 (2.7)	0.703
Previous surgical history	72 (39.6)	888 (29.9)	0.006
Cigarette smoking	39 (21.4)	389 (13.1)	0.002
Alcohol drinking	26 (14.3)	254 (8.6)	0.009
Fracture type			0.015
Femoral neck	78 (42.9)	1545 (52.1)	
Intertrochanteric	104 (57.1)	1420 (47.9)	
Preoperative stay (days)	7.5 ± 6.1	5.3 ± 3.8	< 0.001
Intraoperative bleeding (ml)	293.7 ± 236.6	277.3 ± 277.7	0.435
Intraoperative blood transfusion	55 (30.2)	683 (23.0)	0.026
Surgical duration (minutes)	114.7 ± 41.6	110.0 ± 41.6	0.141
Procedure			0.532
Arthroplasty	66 (36.3)	1144 (38.6)	
Osteosynthesis	116 (63.7)	1821 (61.4)	
ASA			0.001
I–II	74 (40.7)	1571 (53.0)	
III–IV	108 (59.3)	1394 (47.0)	
Anesthesia (general)	126 (69.2)	1687 (56.9)	< 0.001
TP (< 60 g/L)	109 (59.9)	1770 (59.7)	0.959
TC (> 5.8 mmol/L)	3 (1.6)	123 (4.1)	0.095
WBC (> 10 * 10^9^/L)	69 (37.9)	860 (29)	0.011
LYM (< 1.8*10^9^/L)	84 (46.2)	1466 (49.4)	0.389
RBC (< Lower limit)	77 (42.3)	1355 (45.7)	0.372
HGB (< Lower limit)	78 (42.9)	1341 (45.2)	0.533
HCT (< Lower limit)	44 (24.2)	787 (26.5)	0.428
PLT (> 300 * 10^9^/L)	36 (19.8)	364 (12.3)	0.003

*BMI* body mass index, *ASA* American Society of Anesthesiologists, *TP* total protein, *TC* serum total cholesterol, *WBC* white blood cell, *LYM* lymphocyte, *RBC* red blood cell, reference range: Female, 3.5–5.0 * 1012/L; males, 4.0–5.5 * 1012/L, *HGB* hemoglobin, reference range: Females, 110–150 g/L; males, 120–160 g/L, *HCT* hematocrit, reference range: Females, 35–45%; males, 40–50%; PLT, platelet

In the multivariate analysis, age (OR 1.04; 95% CI 1.02–1.06; *P* < 0.001), males (OR 2.27; 95% CI 1.64–3.13; *P* < 0.001), respiratory disease (OR 3.74; 95% CI 2.32–6.04; *P* < 0.001), heart disease (OR 1.68; 95% CI 1.14–2.47; *P* = 0.008), cerebrovascular disease (OR 1.58; 95% CI 1.11–2.27; *P* = 0.012), liver disease (OR 2.61; 95% CI 1.33–5.15; *P* = 0.005), preoperative stay (OR 1.08; 95% CI 1.05–1.11; *P* < 0.001) and general anesthesia (OR 1.61; 95% CI 1.15–2.27; *P* = 0.006) were significantly associated with postoperative pneumonia (Table 2). The H–L analysis showed the good fitness (*X*^2^ = 5.009, *P* = 0.757, Nagelkerke *R*^2^ = 0.153).

**Table 2:** Independent predictors of perioperative pneumonia in the multivariate analysis

Variables	OR	95% CI	*P*
Age (for each year increment)	1.04	1.02–1.06	< 0.001
Sex (males)	2.27	1.64–3.13	< 0.001
Respiratory disease	3.74	2.32–6.04	< 0.001
Heart disease	1.68	1.14–2.47	0.008
Cerebrovascular disease	1.58	1.11–2.27	0.012
Liver disease	2.61	1.33–5.15	0.005
Preoperative stay (for each day prolonged)	1.08	1.05–1.11	< 0.001
General anesthesia	1.61	1.15–2.27	0.006

*OR* odd ratio, *95%CI* 95% confidence interval

## Discussion

Pneumonia, a devastating complication in geriatric patients following hip fracture surgery, often results in seriously adverse outcomes. To our knowledge, the risk factors for postoperative pneumonia in this specific group are still debated. In this study, we incorporated the comprehensive risk factors in a large sample to obtain more reliable and applicable results. We found that the incidence of postoperative pneumonia was 5.8%, and age, sex (males), heart disease, respiratory disease, cerebrovascular disease, liver disease, preoperative stay and general anesthesia were identified as significant factors for postoperative pneumonia.

In this research, age was identified as an independent factor for pneumonia, consistent with most previous findings in previous studies [8, 18]. In a recent research, authors found this relationship was progressive with aging, and the risk was as 2.3, 3.9 and 5.6 times in patients aged 60‒69, 70‒79, ≥ 80 years as in patients aged 50 years [19]. Similarly, another study found that individuals aged 80 years and older had a most strong magnitude of OR of 5.1 as compared with those under 50 years [20]. This could perhaps be related that aging can lead to physiological degeneration of pulmonary function, primarily in the form of decline of breathing strength, lung compliance, cough reflex and respiratory defense, eventually bringing about pneumonia [21, 22]. In addition, aging also leads to impaired response of macrophages to injury repair and regeneration of functional tissue when stimulated by inflammation, aggravating local lung tissue damage, leading to reduced immune function and increased risk of pneumonia [13]. Therefore, elderly patients, especially elder elderly patients > 80 years, should be always aware of the risk of pneumonia, and extracorporeal support and preoperative enhanced breathing exercises along with regimented temperature management are also necessarily warranted.

Although we found male sex having a moderate magnitude (OR, 2.27) of risk of pneumonia, its role was not consistent and the mechanism was also unclear and even controversy. Ekström et al. [23] suggested that male patients have worse preoperative health status and higher comorbidity rate, consequently contributing to a twofold increase in pneumonia in a prospective cohort study, contrasted with our finding that females had more prevalent comorbidities. Another study attributed this difference to the poorer pre-injury status [24], which, however, could not be captured in our study. Other studies also suggested that the overwhelmingly predominated smokers and carriers of tracheobronchial conditions being males are the potential reason for risk of pneumonia, but we found that after adjustment for smoking and respiratory disease the males still showed the independent effect on pneumonia. These findings showed there may be other potential explanations or mechanisms for this association, and future study should elucidate this.

As is well known that comorbidities status would substantially affect the postoperative complications, especially for those experiencing both trauma from a major fracture and subsequent surgery within a rather short period. In this study, we found several individual morbid conditions were associated with postoperative pneumonia: heart disease, respiratory disease, cerebrovascular disease and liver disease. These findings emphasized the clinical importance of inadequate physiologic reserve of organs and the undesirable systemic conditions [25, 26]. It is of particular note that chronic respiration disease, e.g. COPD, should be given special attention in practice due to the highest magnitude of risk for pneumonia and this finding was unsurprisingly consistent in the literature [27, 28]. Anyhow, preoperative medical optimization is most, most important for the prevention of postoperative complications, not merely the pneumonia [29–31].

Early surgery within 48 h and even within 24 h may be a most viable modifiable factor for prevention or reduction the occurrence of bed-rest-dependent complications, primarily pneumonia [32]. Delaying surgery can cause pain, prolonged immobilization and the resultant weaken capacity of discharging phlegm, increasing the risk of developing pneumonia [33]. We found 8% increased risk of postoperative pneumonia by an addition day of delay to surgery, in line with most previous studies [33, 34]. However, due to the setting of tertiary referral trauma center of our institution, it is impractical due to the fact that most patients referred to our institution had experienced 1-day delay, and the overheavy surgical capacity within a short period can post a considerable issue. Recently, increasingly emerging evidences have demonstrated the effectiveness of multidisciplinary approach in improving the surgical management hip fracture in the elderly patients, potentially feasible and practical method to reduce the preoperative waiting time [35, 36]. Therefore, it is still a problem demanding solution for surgical room medical staff and operative surgeons to appropriately arrange such large number of hip fracture patients in as settings as such, and maybe a fast treatment channel for older elderly patients and specifically trained nurses allocated to qualified units can be established.

Anesthesia mode selection was also an essential consideration when managing complex major trauma in orthopedic surgery fields [37, 38]. In the present study, general anesthesia was identified as an independent risk factor for development of pneumonia, consistent with findings of previous studies [20, 39]. This was explained by the general anesthesia being an invasive operation, which may cause damage to the respiratory system, affect the respiratory dynamics and muscle function and therefore reduce lung capacity. Meanwhile, intubation under general anesthesia can also impede the defense function, stimulate the increase of respiratory secretions and finally induce occurrence of pulmonary infection [40]. However, the evidence on anesthesia method affecting the postoperative pneumonia was inconclusive so far and selection of anesthesia was depending on numerous factors including patient comorbidities, anticipated surgical duration and surgical procedure, preference and of anesthesiologists on duty and personal factors from patients and their relatives. Therefore, although identified as a seemly modifiable factor, decision to select an anesthesia technique requires multi-aspect communication and coordination.

The high incidence and mortality of postoperative pneumonia in elderly patients with hip fracture suggest that effective respiratory exercise and muscle exercise are necessary to prevent pulmonary complications and promote postoperative rehabilitation. Notably, physiotherapists play an important role in respiratory exercise for geriatric patients who developed postoperative pneumonia after surgeries of hip fracture (especially in patients received osteotomy procedure) [41]. In a non-randomized, quasi-experimental study, Chang et al. [30] found that the incidence of postoperative pneumonia in patients in the thoracic physical therapy group initiated on the first day after surgery was significantly lower compared to that in the control cohort (5.9% vs. 13.9%). It is worth noting that the rate of decline in muscle mass increases by 0.5–1.0%, and loss of muscle mass can affect the effect of postoperative rehabilitation exercise in elderly patients undergoing hip fracture surgeries [42]. Another study showed that patients with decreased skeletal muscle mass in elderly patients with hip fracture also had a large loss of ADL one year after surgery, which would ultimately affect the postoperative functional recovery of patients [43]. Therefore, elderly patients with hip fracture should cooperate with doctors after surgery to strengthen muscle function exercise to improve the prognosis. We suggest that geriatric patients following hip fracture should actively quit smoking after admission and pay specific attention to oral cleanliness after surgery. Also, patients' swallowing function should be monitored in a timely manner and intensive respiratory management and nutritional support can be implemented as necessary.

## Limitations

The major strengths in this study were collection of numerous exploratory risk factors for analysis of their association with pneumonia in geriatric patients following surgically treated hip fracture in a certainly large sample cohort. However, there were several potential limitations. First, this was a single-center retrospective study, possibly biasing the accuracy in collected data and the results, although we tried our best to solve that including using data-double-entry and cross-check. Second, the primary result of postoperative pneumonia was only limited to those occurring in hospitalization period, which might have led to underestimate of serious adverse complication. Third, the generalizability of our results to other settings still requires further investigation, because of its single-center and tertiary referral trauma center design. Fourth, it is of note that the findings were associative rather than causative relationship and therefore should be interpreted with caution. Fifth, as every other logistic regression analysis, the confounding effect remains an issue, because there are many unmeasured, unconsidered or unquantifiable variables.

## Conclusion

In this study, we found that the incidence of postoperative pneumonia was 5.7%, and age, sex (males), heart disease, respiratory disease, cerebrovascular disease, liver disease, preoperative stay and general anesthesia were identified as significant factors for postoperative pneumonia. These results would be conductive to enhancing the understanding in management of geriatric hip fracture and being informed of the risk factors for postoperative pneumonia.

## Data Availability

All the data used during the current study are available from the corresponding author on reasonable request.

## Abbreviations

BMI: Body mass index
ASA: American Society of Anesthesiologists
TP: Total protein
ALB: Albumin
TC: Serum total cholesterol
TG: Triglyceride
WBC: White blood cell
LYM: Lymphocyte
RBC: Red blood cell
HGB: Hemoglobin
HCT: Hematocrit
PLT: Platelet
OR: Odd ratio
95%CI: 95% Confidence interval
